# Detection of Antimicrobial Resistance of Bacteria *Staphylococcus chromogenes* Isolated from Sheep’s Milk and Cheese

**DOI:** 10.3390/antibiotics10050570

**Published:** 2021-05-12

**Authors:** Ivana Regecová, Jana Výrostková, František Zigo, Gabriela Gregová, Mariana Kováčová

**Affiliations:** 1 Department of Food Hygiene, Technology and Safety, The University of Veterinary Medicine and Pharmacy in Košice, Komenského 73, 041 81 Košice, Slovakia; ivana.regecova@uvlf.sk (I.R.); kovacova@student.uvlf.sk (M.K.); 2 Department of Animal Nutrition and Husbandry, The University of Veterinary Medicine and Pharmacy in Košice, Komenského 73, 041 81 Košice, Slovakia; frantisek.zigo@uvlf.sk; 3 Department of Public Veterinary Medicine and Animal Welfare, The University of Veterinary Medicine and Pharmacy in Košice, Komenského 73, 041 81 Košice, Slovakia; gabriela.gregova@uvlf.sk

**Keywords:** antibiotic, *mecA* gene, MALDI–TOF, PCR, staphylococci

## Abstract

Antimicrobial and multidrug resistance is detected in nonaureus staphylococci, including *Staphylococcus chromogenes*, which commonly causes intramammary infections. Recent clinical studies point to the presence of methicillin-resistant *S. chromogenes.* Therefore, this study aims to determine the prevalence of this species in samples of sheep‘s milk and cheeses made from them. Isolates were identified by polymerase chain reaction and matrix-assisted laser desorption/ionization time-of-flight mass spectrometry (MALDI–TOF). A total of 208 staphylococcal isolates were identified. Of these, 18% were identified as *S. chromogenes*. The antimicrobial resistance of the identified isolates was determined using the agar dilution method against penicillin, ceftaroline, teicoplanin, gentamicin, erythromycin, tetracycline, and ofloxacin. The highest resistance was found to penicillin (95%), tetracycline (86%), and oxacillin (81%). The highest sensitivity was confirmed for gentamicin (55%). The study also confirmed the presence of methicillin resistant staphylococcal isolates (30%) based on the phenotypic manifestation of antimicrobial resistance and detection of the presence of the *mecA* gene. The study shows that the tested isolates (62%) were multidrug resistant. Resistance to two antibiotics was most often found (39%).

## 1. Introduction

Worldwide, sheep‘s milk consumption accounts for 1.3% of world milk production. Sheep’s milk consumption is 10.6 million t/year, and the sheep population is 1.2 billion [1,2]. The market in sheep’s milk products is increasing due to higher product quality, high yield, and nutritional value, because sheep’s milk represents a higher concentration of fats, proteins, vitamins, and minerals compared to the milk of other domestic species [3]. Sheep’s milk is commonly used to make soft cheeses, yogurts, and beverages [4]. However, bacteria of the genus *Staphylococcus*, which are the main cause of mastitis in dairy sheep, are often detected in sheep’s milk. They are responsible for more from 65% of mastitis cases. In ewes, bacterial mastitis is a financially significant problem, especially in dairy type production systems [5]. Although the coagulase positive species (*S. aureus*) is more pathogenic and directly associated with more severe mastitis, nonaureus staphylococci (NAS) are increasingly recognized as the cause of clinical and subclinical mastitis [6]. *Staphylococcus chromogenes* is one of the primary etiological agents of subclinical mastitis [5,7]. Recently, this type of staphylococcus has shown resistance to antibiotics in the dairy industry [6]. 

A high prevalence of ARG has been reported, especially in NAS, highlighting the risk of developing more drug-resistant staphylococci from the ARG reservoir [8].

The development and spread of antibiotic resistance in staphylococci is caused by horizontal gene transfer. In particular, NASs serve as a group of plasmid resistance, and they can significantly contribute to the spread of this resistance [9]. The resistance mechanisms specified by the resistance genes fall into three major categories: (i) enzymatic inactivation, (ii) active efflux, or (iii) protection/modification/replacement of the cellular target sites of the antimicrobial agents. Mobile genetic elements, in particular plasmids and transposons, play a major role as carriers of antimicrobial resistance genes in animal staphylococci. They facilitate the exchange of resistance genes with staphylococci of human origin but also with other Gram-positive bacteria (Wendlandt et al., 2013) [10].

Several reports indicate that NAS are the most prevalent bacteria recovered from subclinical mastitis of sheep and goats [11,12,13,14], thus creating opportunities for crosscolonization and infection among sheep and farmers, due to their antimicrobial resistance and pathogenicity gene pools [15].

In the last decade, a significant increase in antibiotic-resistant infections has been recorded among ovine NAS, especially for beta-lactams and tetracyclines, which are commonly used in veterinary practice for mastitis treatment [16,17]. Two mechanisms confer penicillin resistance in staphylococci, the most common being production of β-lactamase, encoded by the *blaZ* gene. The other mechanism is due to a penicillin-binding protein transpeptidase (PBP2a), encoded by the *mecA* gene [18], which is carried on a mobile chromosomal element, the staphylococcal chromosomal cassette *mec* (SCC*mec*) [19]. SCC*mec* types are defined by the recombinase (*ccr*) gene complex and the class of the mec gene complex [20]. Recently, a novel PBP2a homologue has been described as encoded by *mecC* [21].

The uncontrolled use of methicillin has led to the development of MRS, which represents a risk to public health due to the transmission of these bacteria from animals to humans and vice versa [22], underlining the need to monitor these microorganisms in the dairy industry. MRS strains are a risk to public health due to their opportunistic abilities, and they can cause mastitis, zoonotic infections and act as a reservoir of antimicrobial resistance in dairy farms [23]. Bacteria of the species *S. chromogenes*, which have recently been isolated from frequently consumed milk and fermented dairy products, are also ranked among MRS [24,25]. However, there are few reports on the antimicrobial resistance profile of *S. chromogenes* isolated from dairy products made from sheep’s milk [26].

As the degree of antibiotic resistance in *S. chromogenes* may differ from other staphylococcal species, it is important to identify it precisely. Traditional methods used for the identification and classification of bacteria are currently supplemented by the sequence analysis of small rRNA subunits by polymerase chain reaction (PCR) methods [27]. However, it is necessary to shorten the analysis time in the species identification of staphylococci without any prior knowledge of genetic targets. One such method is a matrix-assisted laser desorption/ionization time-of-flight mass spectrometer (MALDI–TOF MS). Many studies report the fast, cost-effective, and accurate performance of these MALDI–TOF MS systems [28]. 

Based on the above, the aim of our study is to determine the presence of *Staphylococcus chromogenes* by PCR and MALDI–TOF MS in sheep’s milk and sheep’s cheese made from it and subsequently detect antimicrobial resistance and the presence of MRS bacteria of the species *S. chromogenes*.

## 2. Results

**Figure 1:**
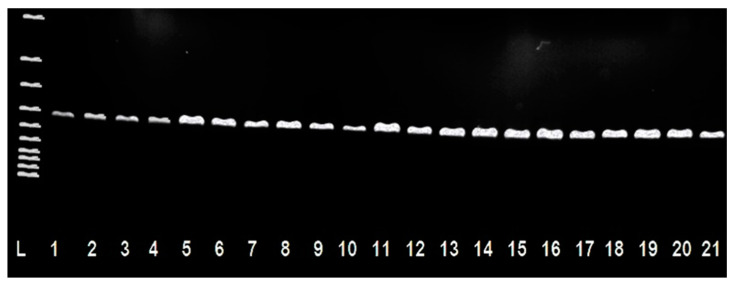
Identification of isolates of *Staphylococcus* spp. from sheep’s milk and cheese using the PCR method (420 bp). L: 100 bp ladder; Line 1: reference strain CCM 4223 *S. aureus*; Lines 2–21: isolates *Staphylococcus* spp.

A total of 208 isolates of *Staphlococcus* spp. were detected by a microbiological culture examination of individual samples of sheep’s milk and sheep’s cheese and identification of the isolates by PCR (Figure 1). Of these, 116 strains were isolated from raw sheep’s milk samples, and 92 strains were isolated from sheep’s cheese samples made from the sheep’s milk examined.

Subsequently, *S. chromogenes* species (37 isolates) were identified by MALDI–TOF MS. The score value of the isolates thus identified ranged from 2.198 to 2.279, which represents an accurate identification at the species level. This species represented 18% of all isolated staphylococcal strains in this study. Based on species identification of the isolated strains, a higher prevalence of *S. chromogenes* was detected in the raw sheep’s milk samples (20%; 23 isolates) compared to the samples made from sheep cheese (15%; 14 isolates). 

Because raw sheep’s milk can be a good substrate for the growth of resistant staphylococci, we also examined the identified isolates in terms of their resistance to selected antibiotics. Overall, very frequent resistance to penicillin (95%; 35 isolates), tetracycline (86%; 32 isolates), and oxacillin (81%; 32 isolates) was detected in the *S. chromogenes* isolates we identified. The minimal inhibitory concentration (MIC) of penicillin-resistant isolates ranged from 0.25 to 0.5 mg/L. For tetracycline resistance, the MIC was set at a cut-off value of 16.0 and 32.0 mg/L. Oxacillin-resistant strains had an MIC determined from 0.5 to 1.0 mg/L. For the antibiotic oxacillin (OX) at a concentration of 0.5 mg/L, out of 37 isolates (*S. chromogenes*), 25 isolates are resistant to the selected antibiotic. In percentages, it is more than 67%. At a concentration of 1 mg/L, 5 isolates grew, which represents 13.5%. At twice the concentration of the antibiotic OX, 20 isolates were more resistant. The odds ratio is 13.33. The chance that the isolate with the OX antibiotic and 0.5 mg/L dose is resistant is 13.33 higher than the growth with the higher 1 mg/L dose. The 95% confidence interval for the odds ratio is 4.15–42.84. This means that the chance that the isolate is resistant at a dose of 0.5 mg/L is with a 95% probability 4.15–42.84 times higher than that the isolate is resistant at a dose of 1 mg/L. The *p* value is significantly lower than the alpha, and this difference is statistically significant (*p* < 0.001). 

For the antibiotic ofloxacin (OFX) and its concentration of 4 mg, 10 isolates were resistant. At a concentration of 8 mg/L, only 2 isolates were resistant. The odds ratio (OR) is 6.48. There is a 6.48 times higher chance that an isolate shows resistance at a dose of 4 mg/L than that it is resistant at a dose of 8 mg/L. The difference is significant at the alpha (*p* < 0.05) significance level. The differences for all other antibiotics and doses are not statistically significant. (Table 1).

In contrast, high sensitivity (55%) was observed in this species, especially for gentamicin and ofloxacin (54%). The MICs of gentamicin-sensitive isolates were determined from 2.0 to 4.0 mg/L. For ofloxacin-susceptible strains, the MIC was 0.5–1.0 mg/L. Intermediate sensitivity was also confirmed, which was most frequently recorded for teicoplanin in 49% of strains isolated from sheep’s milk and 35% of strains isolated from sheep’s cheese (MIC was 16 mg/L). In particular, raw sheep’s milk isolates showed the most common resistance to penicillin, tetracycline, oxacillin, and erythromycin (Table 2). These detected resistances were above 40%. *S. chromogenes* strains isolated from sheep cheese samples showed the most common resistance to penicillin, oxacillin, erythromycin, and tetracycline. This resistance was above 30% (Table 2). 

**Figure 2:**
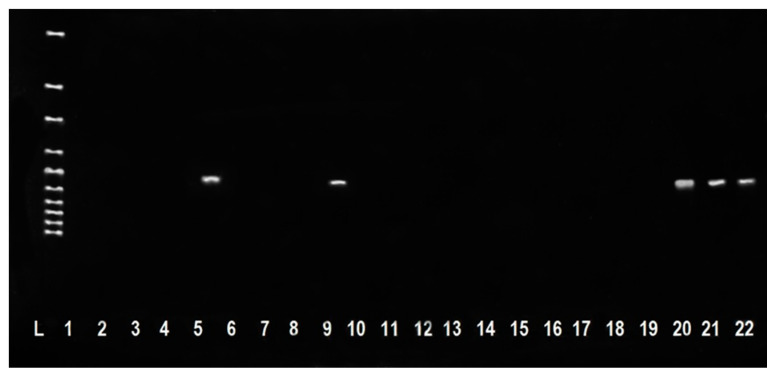
Detection of *mecA* gene in *S. chromogenes* isolates isolated from sheep’s cheese (527 bp). L: 100 bp ladder; Line 1: water (negative control); Lines 5,9,20,21: isolates *S. chromogenes* with *mecA* gene; Lines 2, 3, 4, 6, 7, 8, 10, 11, 12, 13, 14, 15, 16, 17, 18, 19: isolates *S. chromogenes* without *mecA* gene; Line 22: reference strain CCM 4750 *S. aureus* (positive control).

In our study, strains that showed simultaneous resistance to penicillin and oxacillin were confirmed. As is well known, resistance to a wide range of β-lactam antimicrobials can be deduced from testing only penicillin and oxacillin (or cefoxitin). In addition, the presence of oxacillin-resistant strains indicates the presence of methicillin-resistant staphylococci. This resistance is most often encoded by the *mecA* gene [9,29]. It is for these reasons that the determination of resistance to penicillin and oxacillin, and at the same time, the detection of the *mecA* gene in the isolates of *S. chromogenes* identified by us were started (Figure 2). The presence was confirmed in 11 strains (7 isolates from sheep’s milk and 4 isolates from sheep’s cheese), which at the same time showed resistance to both oxacillin and penicillin. Based on the results of our study, these strains were considered MRS isolates. 

When examining *S. chromogenes* isolates, multidrug resistance was also confirmed in 62% (23 isolates) of all tested isolates. Strains isolated from sheep’s cheese showed a higher percentage of multidrug resistance (71%) than strains isolated from sheep’s milk (57%). 

Resistance to two antibiotics simultaneously was most often detected (39%). In isolates of *S. chromogenes* from sheep’s milk and sheep’s cheese, resistance to 6 antibiotics simultaneously was also confirmed (Table 3), of which in two isolates, the presence of *mecA* gene was also detected.

## 3. Discussion

The impact of resistant CoNS on animal health in the dairy industry and its subsequent spread in the food chain have received more attention in the last decade [30]. This is also confirmed by the study of [31], in which out of a total of 64 evaluated samples, the presence of staphylococci was identified in 33 samples using phenotypic assays. CoNS were originally referred to as one large, uniform group of bacteria [30]. However, thanks to advances in molecular identification techniques, individual types of CoNS are easier to identify and study [32,33]. Some types of CoNS are so-called environmental CoNSs and include, but are not limited to, *Staphylococcus equorum* and *Staphylococcus fleurettii* [34]. At the other end of the spectrum are the so-called host-adapted species of CoNS, including *S. chromogenes* [35], because it is the predominant species of CoNS, which is found mainly in milk [24,34]. Its presence in the last period, especially in sheep’s milk, was confirmed by Zigo et al. and Virdis et al. in more than 7% of the tested CoNS [36,37]. The detection of *S. chromogenes* in ripening cheeses was also confirmed in the study by El-Sharoud et al., where out of the total number of 87 identified staphylococci, 6% were *S. chromogenes* [38]. The presence of *S. chromogenes* isolates (0.5% of 431 isolates) in sheep cheese was also confirmed by Coton et al. [39]. Our findings are consistent with previous studies, where *S. chromogenes* was identified in sheep milk samples and cheese.

Of the total population of isolated staphylococci, *S. chromogenes* accounted for 18%. Specifically, in raw sheep’s milk, it was identified in 20% of the total population and in 15% of the total population of staphylococci isolated from sheep cheese. A higher percentage of *S. chromogenes* in sheep cheeses was also found by Rahmdel et al., who confirmed the presence of up to 39% staphylococci by PCR method [40]. 

In addition to the pathogenicity of *Staphylococcus chromogenes*, its antimicrobial resistance is also a serious problem, as the genetic determinants of resistance are transmitted to various bacterial strains and species through the exchange of genetic material. Antimicrobial-resistant staphylococcal strains are detected in animals and can be transmitted to foods of animal origin [17,41]. This is also confirmed by a study by Seng et al., who reported up to 80% resistance to several types of antibiotics in staphylococcal isolates from dairy products [42]. Moreover in our study, resistance to at least one investigated antibiotic was confirmed in 95% of isolates. Resistance to penicillin and oxacillin was confirmed in these isolates, similar to the study by Zigo et al., where the resistance of staphylococci isolated from sheep’s milk to penicillin was 51.4% [36]. Resistance to β-lactam antibiotics in staphylococcal isolates was also confirmed by Sampimon, found that 18% of *S. chromogenes* isolates were resistant to penicillin [43]. Similar rates of β-lactam antibiotic resistance in *S. chromogenes* (18%) were also reported in a US study [44]. Persson-Waller et al. also performed the detection of resistance in *S. chromogenes* (33%) to β-lactam antibiotics in their study [24]. Recently, attention has been focused mainly on resistance to penicillin-stable penicillins, which is referred to as “methicillin resistance” or “oxacillin resistance”. Most resistance to methicillin (oxacillin) is mediated by *mecA*-encoding PBP2a. Isolates in which the presence of the *mecA* gene is confirmed should be classified as resistant to methicillin (oxacillin) according to CLSI [45]. MRS isolates, defined by the agar dilution method as oxacillin-resistant, are considered resistant to other β-lactam substances, penicillins, cephems (excluding sephtharoline), and carbapenems. This is because most cases of documented MRS infections have responded insufficiently to treatment with β-lactam antibiotics or because convincing data documenting the clinical efficacy of these agents have not been provided [46]. 

In this study, we have identified 30 “resistant” isolates of *S. chromogenes* at or above the breakpoint of 0.5 μg/mL oxacillin, but we have only identified the *mecA* gene in 11 of these isolates. This is also indicated by the different results phenotypic methods based on clinical breakpoints that require an increase in MIC above a threshold to define resistance, and the presence of genetic determinants of antimicrobial resistance reflects acquisition of a mechanism that may either be sufficient to cause resistance or simply enhance bacteria survival in the presence of low concentrations of antimicrobials [47]. Tests for *mecA* or for the protein encoded by *mecA*, PBP2a, are the most accurate methods for prediction of resistance to oxacillin and could be used to confirm results of staphylococci isolates from serious infections [45]. Oxacillin is no longer the agent recommended by the Clinical and Laboratory Standards Institute for CoNS phenotypic tests to predict resistance to penicillinase-stable penicillins. Antimicrobial susceptibility tests using oxacillin are often difficult to interpret, despite changes that have improved discrimination between oxacillin-susceptible and -resistant strains. Minimum inhibitory concentration panels in which oxacillin is tested must be examined carefully to detect any growth that may be indicative of resistance [48,49,50]. Several groups of investigators [51,52,53] have reported that the results of cefoxitin tests correlate better with the presence of *mecA* than do the results of tests using oxacillin, and the test is now the preferred method for testing coagulase negative staphylococci [48]. Although there are non-*mecA* gene-mediated mechanisms of MRS, they are very rarely encountered in clinical practice. A more likely explanation is that there could be some minor variations in the *mecA* gene near the region of primer annealing leading to negative PCR results, although functionally, the gene may be unaffected. Such an occurrence can be confirmed using multiple primers targeting different regions of the *mecA* gene [54]. In addition to *mecA*, other mec genes, such as *mecB* and *mecC*, have also been recognized in association with β-lactam resistance in staphylococci [55,56]. The *mecB* and *mecC* genes have usually been identified within mobile genetic elements (MGEs) that were similar to *SCCmec* [57]. Several studies have reported that these *SCCmec* elements can be transferred between CoPS and CoNS isolates [58,59].

The importance of detecting MRS isolates of *S. chromogenes* in the agrofood chain also stems from the possibility of zoonotic infection in consumers and workers involved in animal husbandry and food production of animal origin [17,60]. Regarding food, MRS isolates have been identified in samples from food processors and in milk and dairy products in several studies around the world. For example, in Turkey, 30% of milk samples, 18% of cream, and 34% of cheese samples were contaminated with MRS [61], and in Mexico, MRS was found in 18.1% of handmade cheese samples [62]. Due to the frequent occurrence of MRS strains in the production of sheep’s milk and milk products, their elimination is necessary. The most important measure in terms of ensuring the reduction of MRS in sheep’s milk and dairy products is pasteurization. Other measures to reduce MRS in milk and cheese include good hygiene practices on farms, from primary teat disinfection combined with good milking hygiene to the final processing of milk and dairy products [63].

Groups of animal and human staphylococci share a large number of resistance genes, and only a relatively small number of resistance genes have been found exclusively in human or animal staphylococci. Although, host adaptation at the strain level is known [64,65,66]. In the study by Martins et al., the presence of *S. chromogenes* isolated from sheep’s milk was confirmed [14]. Antimicrobial resistance and *mecA* gene detection in the 10 drugs tested were effective against most CNS samples in an in vitro antimicrobial susceptibility test. Samples were resistant to penicillin (17.0%), tetracycline (10.7%), clindamycin (6.25%), erythromycin (2.7%), cotrimoxazole (2.7%), gentamycin (0.9%), ciprofloxacin (0.9%), and rifampicin (0.9%). All samples were sensitive to vancomycin and linezolid. Specifically, in *Staphylococcus chromogenes*, 12.5% of isolates were detected as resistant to PEN and E.

Recent evidence from around Europe does not indicate significant problems of resistance to antibiotics commonly used for cases of mastitis in sheep. Vautor et al. reported only sporadic resistance in *S. aureus* isolated in France [67]. Onni et al. in Italy also found limited resistance in *S. epidermidis*, except to penicillin, for which the resistance rate was 38% [16]. Similar results have been observed in Turkey, where in coagulase-negative isolates from subclinical mastitis only resistance to β-lactams was noteworthy (43%), whilst there was much smaller frequency of resistance to tetracycline (11%) and even less to other agents [68]. Further work in Turkey corroborated those findings, the rate of resistance to penicillin was 27% and to tetracycline 8% [69]. Martins et al. published similar results: 17% of isolates were resistant to penicillin and 11% to tetracycline [14]. Finally, evidence from Greece was consistent with the above, as the frequency of resistant isolates was <25% for all the antimicrobial agents evaluated [70]. Different findings have been reported by Azara et al., who found greater resistance to tetracycline (50%) of *S. aureus* from clinical mastitis [71]. In contrast to the above results, in Brazil, Franca et al. determined a higher resistance to amoxicillin, erythromycin, lincomycin, streptomycin, and tetracycline (>35% of staphylococcal isolates tested) [72].

In addition to resistance to β-lactamates, resistance to tetracycline (86%) and erythromycin (78%) was also detected to a greater extent in the isolates we examined. Resistance to erythromycin and tetracycline in *S. chromogenes* was also detected by Lüthje et al. and Lee et al. [73,74]. However, in a study by Devries et al., out of 73 staphylococcal strains from different dairy farms, 70 strains of *S. chromogemes* sensitive to neomycin, gentamicin, erythromycin, enrofloxacin, and penicillin were detected [75]. The final result was confirmed by the absence of the *mecA* gene in each of the 13 strains in which this gene was detected. Whereas in Flanders, Belgium, Catry et al. identified *Staphylococcus chromogenes* in subclinical mastitis from 44 dairies [76]. Of the 24 strains, resistance to β-lactam antibiotics was confirmed in two isolates. In these isolates, the presence of the *mecA* gene was confirmed. Kenar et al. collected a total of 572 positive milk samples from 18 private farms in midwestern Anatolia [77]. Of these, 67 CONS strains were confirmed. The most frequently identified species were *Staphylococcus epidermidis* (26.8%), *Staphylococcus simulans* (20.8%) followed by *Staphylococcus warneri* (14.9%), and in 6%, *Staphylococcus chromogenes* was also confirmed. The most resistant isolates were trimethoprim + sulfametoxazol (76.2%), erythromycin (73.2%), oxacillin and ampicillin (70.2%), followed by penicillin (58.3%), gentamicin (53.8%), tetracycline (52.3%), vancomycin (51.8%), ciprofloxacin (26.9%), cefoxitin (23.9%), and cephalothin (13.5%). These results suggest that CONS species are highly resistant concentrations to beta-lactam antibiotics, which are extensively used in the prevention and treatment of mastitis without any antibiotic susceptibility test in Turkey. Phophi et al. detected 93% of *S. chromogenes* resistant to at least one antibiotic investigated, similar to our study [78]. The isolates were mainly resistant to penicillin (63%) and erythromycin (54%). Furthermore, *S. chromogenes* showed 52% concomitant resistance to more than one antibiotic at a time. The most common resistant pattern among *S. chromogenes* was the penicillin–ampicillin–erythromycin sample (91%). In our study, the most frequently resistant formula of penicillin–oxacillin–tetracycline was confirmed in multidrug-resistant strains. 

Nunes et al. also proved multidrug resistance in 14 coagulase-negative staphylococci (including *S. chromogenes*) out of 19 strains [79]. They were resistant to β-lactams and vancomycin, corresponding to 73% of the total isolates identified, while nine isolates (64%) were resistant to tetracycline and gentamicin, and eight isolates (57%) were resistant to neomycin, erythromycin, and chloramphenicol. The multiresistance of *S. chromogenes* isolates detected in our study is consistent with previous studies that reported the presence of resistant and multiresistant staphylococcal strains in raw milk and dairy products [80,81].

Antimicrobial resistance genes in staphylococcal strains form a risk to public health because they increase the gene pool from which pathogenic bacteria can capture genetic determinants of resistance. Foods can be contaminated with antimicrobial-resistant bacteria and their genetic determinants in several ways: by the presence of antibiotic-resistant bacteria in foods selected using antibiotics during agricultural production; by the presence of resistance genes in bacteria added during food processing (starter cultures and probiotics); and by crosscontamination with antimicrobial-resistant bacteria during food processing. At the same time, raw food products can be consumed without prior processing or preservation and therefore pose a significant risk of transmitting antimicrobial resistance to humans, as ultimately the resistant bacteria present are not killed [80]. For this reason, raw sheep’s milk and milk products may be potentially contaminated with such bacteria that survive the production process and subsequently introduce genetic determinants of resistance in the gastrointestinal tract of the consumer by horizontal transmission of the intestinal microflora [81]. Antibiotic resistance genes can also be transferred by gene transfer mechanisms (HGT) from bacteria of the microflora of the gastrointestinal tract to clinical pathogens, leading to the acquisition of resistance in recipients of clinical strains [82,83]. Mutations at a common cellular target and acquiring a general drug efflux pump can lead to resistance to several antibiotics. In the case of coselection, the microbe may become resistant to several different antibiotics, each with distinct mechanisms of action due to the same or related genetic determinant(s) [84].

## 4. Materials and Methods

This study was carried out in the period from April to September 2020. Fresh unpasteurized sheep’s milk from Wallachian ewes and cheese made from the examined milk were obtained from two farms in the border area of Slovakia and Hungary in the Slanské vrchy region. From each farm, five samples of milk from early milking were taken from which cheeses were subsequently produced without the addition of cheese starter culture, in the traditional way of production [85]. Milk was obtained by hand milking in accordance with hygienic conditions. Milk samples and cheese made from them were placed in sterile sample containers and transported at 4 °C without the addition of preservatives to the laboratory at the University of Veterinary Medicine and pharmacy in Košice for analysis. Within 4 hours of collection, samples of raw milk and cheeses made from it were subjected to microbiological analysis.

### 4.1. Isolation of Staphylococci

Ten samples of sheep’s milk were used for microbiological examinations. Subsequently, sheep’s cheeses were made from the examined milk without the addition of a starter culture. From these cheeses, after a short maturation period (3 days), 10 samples were taken from individual cheeses for microbiological examination. A stock suspension and further 10-fold dilutions were prepared from the test samples according to ISO standard [86]. Staphylococcal isolates were subsequently isolated from the examined samples according to ISO standard [87]. After isolation, individual strains were identified by PCR and MALDI–TOF MS.

### 4.2. Identification of Isolates

Total genomic DNA was isolated according to Hein et al. [88]. The obtained supernatant was used as a source of DNA in PCR reactions according to Strommenger et al. [89] using primers 16S1 (CAGCTCGTGTCGTGAGATGT) and 16S2 (AATCATTTGTCCCACCTTCG) (Amplia s.r.o, Bratislava, Slovakia). FIREPol^®^ MasterMix (Solis Biodyne, Tartu, Estonia) was used for the PCR reaction. In a total volume of 20 µL containing 5 ng/μL of template DNA and 10 pmoL of each of the primers. The mixture was heated to 95 °C for 5 min during the initial denaturation 30 amplification cycles (denaturation 95 °C/30 s, annealing 55 °C/30 s with extension 72 °C/2 min) were performed in a thermocycler (TECHNE TC-512, London, UK) with a final extension of 10 min/72 °C. 

Species identification of *S. chromogenes* isolates was performed by MALDI–TOF MS according to the standard Bruker Daltonics protocol [90]. The analysis of the results was performed in an Ultraflex III instrument. The obtained results were processed using Flex Analysis software, version 3.0, and evaluated using BioTyper software, version 1.1 (Bruker Daltonics, Billerica, MA, USA).

The identity of the PCR products with the selected primers was confirmed by a commercial company (GATC Biotech AG, Cologne, Germany). The DNA sequences obtained from isolates were searched for similarity to those available at the GenBank–EMBL (the European Molecular Biology Laboratory) database using the BLAST program (NCBI software package).

### 4.3. Detection of Antimicrobial Resistance

The identified *S. chromogenes* isolates were tested for antibiotic susceptibility using the agar dilution method (ADM) according to the procedure described in CLSI document [46]. Test plates with a final concentration of antibiotics (Merck KGAA, Darmstadt, Germany) were used to determine the minimum inhibitory concentration (MIC): penicillin (PEN) 0.06; 0.12; 0.25; 0.5 mg/L; oxacillin (OX) 0.12; 0.25; 0.5; 1.0 mg/L; teicoplanin (TEC) 4.0; 8.0; 16.0; 32.0; 64.0 mg/L; gentamicin (GN) 2.0; 4.0; 8.0; 16.0; 32.0 mg/L; erythromycin (E) 0.25; 0.5; 1.0; 2.0; 4.0; 8.0; 16.0 mg/L; tetracycline (TE) 2.0; 4.0; 8.0; 16.0; 32.0 mg/L; and ofloxacin (OFX) 0.5; 1.0; 2.0; 4.0; 8.0 mg/L.

To confirm MRS strain, the presence of the *mecA* gene was detected by PCR according to Poulsen et al. [91]. The primers used to confirm the presence of the *mecA* gene were MecA1 (GGGATCATAGCGTCATTATTC) and MecA2 (AACGATTGTGACACGATAGCC) (Amplia s.r.o, Bratislava, Slovakia). The PCR reaction conditions were the same as for the genus identification of staphylococci mentioned above. The size of the final product of this amplification was 527 bp. 

*S. aureus* CCM 4750 (Czech Collection of Microorganisms, Brno, Czech Republic) was used as a reference strain for PCR and ADM in this study. PCR water (Top–Bio, Vestec, Czech Republic) without template was used as a negative control in each PCR reaction.

The identity of PCR products with the selected primers was confirmed by a commercial company (GATC Biotech AG, Cologne, Germany). The DNA sequences obtained from isolates were searched for similarity to those available at the GenBank-EMBL database using the BLAST program (NCBI software package).

### 4.4. Statistical Analysis

An odds ratio (OR) test was applied for a significant differences analysis of isolates of *S. chromogenes* antibiotic resistance. A confidence interval set to *p* < 0.05 at 95% was conducted with MedCalc Statistical Software version 19.2.6 (MedCalc Software bv, Ostend, Belgium).

## 5. Conclusions

Our study confirmed the presence of resistant, multiresistant strains and methicillin-resistant strains of *S. chromogenes* in sheep’s milk and cheeses made from unpasteurized sheep’s milk. The results of the study point to the need to examine not only the most important species of *S. aureus* and *S. epidermidis* in sheep’s milk and dairy products but also other species of CoNS, among which we include *S. chromogenes*. The conclusions of the study confirm the need for further investigation of the epidemiology and genetic diversity of methicillin-resistant *S. chromogenes* in the agrofood chain, especially in the processing of milk and dairy products regarding food safety. Therefore, it is necessary to examine in detail the mechanisms of development and transfer of determinants of resistance, the rational use of antibiotics, and the development of new, more effective antibiotics, as well as the regular monitoring of existing antibiotic resistance. Strains of *S. chromogenes* from sheep’s milk are among the little-studied species in terms of antimicrobial profile. The study was intended to contribute to the convergence of knowledge in a selected geographical area where there is little infrastructure and high demand for sheep’s milk products. Although several studies have been performed on the presence of resistant isolates in sheep’s milk, there is still insufficient information on local studies. 

## Figures and Tables

**Table 1:** MIC determination of *S. chromogenes* using agar dilution method.

Strains	MIC (mg/L)											
	ATB	0.12	0.25	0.5	1	2	4	8	16	32	OR	*p* Value
*Staphylococcus chromogenes* (*n* = 37)	PEN	2	19	16	-	-	-	-	-	-	1.3854	0.4853
	OX	2	5	25 ^a^	5 ^b^	-	-	-	-	-	13.3333	<0.0001
	TEC	-	-	-	-	-	2	3	32	-	-	-
	GN	-	-	-	-	11	11	9	5	1	5.6250	0.1237
	E	-	1	3	1	1	2	18	11	-	2.2392	0.0981
	TE	-	-	-	-	1	3	1	15	17	0.8021	0.6390
	OFX	-	-	7	13	5	10 ^a^	2 ^b^	-	-	6.4815	0.0220

ATB: antibiotics. ^a, b^: means within a row different superscript differ (*p* < 0.05). MIC: minimal inhibitory concentration. PEN: penicillin. OX: oxacillin. TEC: teicoplanin. GN: gentamicin. E: erythromycin. TE: tetracycline. OFX: ofloxacin. Gray color represents the breakpoint that categorizes staphylococci as “resistant “.

**Table 2:** Number of susceptible (S), intermediate sensitive (I), and resistant (R) isolates of *S. Chromogenes*.

*S. chromogenes*		Antibiotics							
			PEN	OX	TEC	GN	E	TE	OFX
sample of sheep’s milk	(n = 23)	S	2	5	4	15	3	3	15
		I	0	0	18	4	3	0	2
		R	21	18	1	4	17	20	6
sample of sheep’s cheese	(n = 14)	S	0	2	1	7	1	1	5
		I	0	0	13	5	1	1	3
		R	14	12	0	2	12	12	6

n—number isolates, PEN—penicillin, OX—oxacillin, TEC—teicoplanin, GN—gentamicin, E—erythromycin, TE—tetracycline, OFX—ofloxacin.

**Table 3:** Identified phenotypes of resistance of staphylococcal isolates to several antibiotics simultaneously.

Resistance Phenotype	Sheep’s Milk	Sheep’s Cheese
PEN-E	1	0
PEN-TE	2	1
PEN-OX	3 *	2 *
PEN-OX-E	0	2
PEN-OX-TE	3 *	0
PEN-OX-TE-E	2	3
PEN-OX-TE-E-GN	0	1 *
PEN-OX-TE-E-OFX	1	0
PEN-OX-TE-E-GN-OFX	1 *	1 *
∑	13	10

∑: summary. *: isolates with *mecA* gen. PEN: penicillin. OX: oxacillin. TEC: teicoplanin. GN: gentamicin. E: erythromycin. TE: tetracycline. OFX: ofloxacin.
